# Case analysis of long-term negative psychological responses to psychedelics

**DOI:** 10.1038/s41598-023-41145-x

**Published:** 2023-09-25

**Authors:** Rebecka Bremler, Nancy Katati, Parvinder Shergill, David Erritzoe, Robin L. Carhart-Harris

**Affiliations:** 1 Centre for Psychedelic Research, Imperial College London, London, UK; 2 Psychedelics Division, Neuroscape, University of California San Francisco, San Francisco, USA

**Keywords:** Risk factors, Drug regulation, Public health

## Abstract

Recent controversies have arisen regarding claims of uncritical positive regard and hype surrounding psychedelic drugs and their therapeutic potential. Criticisms have included that study designs and reporting styles bias positive over negative outcomes. The present study was motivated by a desire to address this alleged bias by intentionally focusing exclusively on negative outcomes, defined as self-perceived ‘negative’ psychological responses lasting for at least 72 h after psychedelic use. A strong justification for this selective focus was that it might improve our ability to capture otherwise missed cases of negative response, enabling us to validate their existence and better examine their nature, as well as possible causes, which could inspire risk-mitigation strategies. Via advertisements posted on social media, individuals were recruited who reported experiencing negative psychological responses to psychedelics (defined as classic psychedelics plus MDMA) lasting for greater than 72 h since using. Volunteers were directed to an online questionnaire requiring quantitative and qualitative input. A key second phase of this study involved reviewing all of the submitted cases, identifying the most severe—e.g., where new psychiatric diagnoses were made or pre-existing symptoms made worse post psychedelic-use—and inviting these individuals to participate in a semi-structured interview with two members of our research team, during which participant experiences and backgrounds were examined in greater depth. Based on the content of these interviews, a brief summary of each case was compiled, and an explorative thematic analysis was used to identify salient and consistent themes and infer common causes. 32 individuals fully completed an onboarding questionnaire (56% male, 53% < age 25); 37.5% of completers had a psychiatric diagnosis that emerged *after* their psychedelic experience, and anxiety symptoms arose or worsened in 87%. Twenty of the seemingly severer cases were invited to be interviewed; of these, 15 accepted an in-depth interview that lasted on average 60 min. This sample was 40% male, mean age = 31 ± 7. Five of the 15 (i.e., 33%) reported receiving new psychiatric diagnoses after psychedelic-use and all fifteen reported the occurrence or worsening of psychiatric symptoms post use, with a predominance of anxiety symptoms (93%). Distilling the content of the interviews suggested the following potential causal factors: unsafe or complex environments during or surrounding the experience, unpleasant acute experiences (classic psychedelics), prior psychological vulnerabilities, high- or unknown drug quantities and young age. The current exploratory findings corroborate the reality of mental health iatrogenesis via psychedelic-use but due to design limitations and sample size, cannot be used to infer on its prevalence. Based on interview reports, we can infer a common, albeit multifaceted, causal mechanism, namely the combining of a pro-plasticity drug—that was often ‘over-dosed’—with adverse contextual conditions and/or special psychological vulnerability—either by young age or significant psychiatric history. Results should be interpreted with caution due to the small sample size and selective sample and study focus.

## Introduction

In recent decades, we have witnessed the publication and promotion of promising research results regarding the therapeutic potential of psychedelic drugs, particularly in relation to psychedelic-assisted therapy in the treatment of mental illness^1–6^. Psychedelics are, however, much more commonly used outside of research settings. Thus, the emphasis on positive outcomes in controlled studies may present a misleadingly, over-generalized positive picture of the effects of psychedelics.

There is a growing global industry of illegal, semi-legal and fully legal psychedelic retreats and care services, particularly in Europe and the Americas. Moreover, evidence suggests that an increasing number of individuals struggling with mental health conditions are seeking to self-medicate with psychedelics^7^ and epidemiological data suggests a sizeable increase in the prevalence of psychedelic-use in the last 15 years^8–10^. Questionnaire based sampling of naturalistic use of psychedelics have tended to yield positive findings about the mental health benefits vs. risks [e.g.,^12–15^]; however, self-selection and confirmation biases may have skewed findings in this direction. Moreover, this approach may inadvertently under-sample underwhelming or iatrogenic responses e.g., due to attrition biases [although see^16^].

The high rate and level of promissory messaging linked to the therapeutic potential of psychedelics and raised awareness of this topic e.g., due to impactful journalism^4^, as recently received a counteraction in the form of more critical perspectives^17^ and some mainstream media productions have also taken a predominantly negative focus on psychedelic medicine, claiming that the risk of negative responses is under-represented^18^.

Recognising that cultural and sub-cultural biases in favour of the positive effects of psychedelics may have contributed to positive research outcomes e.g., via self-selecting recruitment and confirmation biases, here we also sought to counter such potential biases by designing a study that explicitly and exclusively focuses on *negative* responses to psychedelics. We chose to focus on negative psychological outcomes and specifically those that endure for greater than 72 h post use—i.e., what we refer to as ‘long-term negative psychological responses’.

Unpleasant acute psychological experiences under psychedelics are not rare—even in research environments. For example, one notable study reported an approximately 40% prevalence of moderate to severe anxiety, panic or distress with high dose psilocybin in healthy volunteers^19^. Colloquially, these experiences are referred to as ‘bad trips’ but more formally, they are defined and measured as ‘challenging experiences’^20^. It is presently unclear, however, how these experiences relate to long-term psychological outcomes. For example, some evidence^21–23^—and reasoning—suggests they may not necessarily foreshadow a worsening of mental health outcomes. Indeed, so-called ‘emotional breakthrough experiences’ under psychedelics^24^, robustly and reliably predict improvements in mental health outcomes across studies and samples^14,25^ and yet these breakthroughs typically feature some degree of resistance of, or struggle with, aversive psychological states.

Given the ambiguous impact of challenging acute psychedelic experiences, here we sought to focus on negative mental health presentations arising or worsening *after* psychedelic use. The prevalence of mental health iatrogenesis post psychedelic use appears to be low but not negligible^26–28^. Prevalence does appear to be lower in controlled research studies. Moreover, consistent with long-held assumptions^29^, iatrogenic responses appear to be highly—if not entirely—context dependent^25,30^.

The importance of ‘set and setting’ for determining responses to psychedelics was first outlined by Leary and colleagues in 1963^29–31^ where ‘set’ refers to psychological factors brought to the experience by the experiencer, and ‘setting’ refers to the immediate environmental context the experiencer finds themselves in. These constructs were usefully expanded on by Betty Eisner in 1997, who emphasised the role of the psychosocial ‘matrix’ in shaping long-term responses to psychedelics^32^. Baseline characterological and mental health presentation are also likely to be important predictors of response^33^. For example, some personality types and psychiatric disorders could be contraindications for psychedelic use in general—as well as for most standard psychedelic therapy approaches^34^.

Low prevalence does not equal low relevance, however, as rare but severe cases will still negatively impact individual lives—with reverberations for family members and others^35,36^. In addition, adverse reactions to psychedelics could affect the success of medical development initiatives involving psychedelics, especially given the politically divisive history associated with these compounds^37,38^. The present approach of focusing on severe long-term negative psychological responses can be seen as consistent with `extreme values analysis` in science^39^ and could motivate further and greater efforts to better understand—in order to prevent—rare, but important, negative psychological responses to psychedelics.

The present study used a two-phase design featuring: (1) an online questionnaire and (2) semi-structured interview approach, to examine cases of negative psychological responses to psychedelics with the aim of better understanding their nature and why they might have occurred. Of particular interest were cases of emerging or worsening psychiatric symptoms including e.g., any cases of psychotic symptoms or symptoms of hallucinogen persisting perceptual disorder (HPPD)^40^. The prevalence of both symptom types is unknown but thought to be low^41,42^. However, there is a history of claiming that psychedelic-use can be directly causal of their emergence^42^. Here, we define ‘psychedelics’ as “LSD, psilocybin/magic mushrooms/truffles, DMT, ayahuasca, 5-MeO-DMT, mescaline (synthetic or plant derived), or the psychedelic-like drug MDMA/ecstasy”.

Brief summaries of each interviewed participants´ case were compiled, and as an exploratory thematic analysis was performed on the interview data. By using case reports and qualitative methods, we sought to gain a richer understanding of psychological and circumstantial complexities and nuances of long-term negative psychological responses to psychedelics that could be easily overlooked and only superficially understood by quantitative methods alone^43^.

## Methods

### Participants, recruitment and procedures

The study was advertised on social media; including Reddit.com (where a link to the questionnaire was shared on fora dedicated to individuals experiencing HPPD after psychedelic use—r/HPPD, r/AyahuascaRecovery), and Twitter (where the link was shared by individuals and organisations with a large number of followers, e.g., Michael Pollan, Tim Ferris, MAPS). A link to the survey was also shared on the platform surveycircle.com. The study researchers directly involved in this study asked people known to them who they thought may be interested; one participant, who completed both phases of the study, was known to the first author. Potential participants were directed to an online survey, which was available from November 2021 until April 2022 for self-selected participation. Participants who wished to participate in an interview were required to submit an email address.

After reviewing submitted forms and selecting cases that appeared to be the most relevant to our interests e.g., cases of emerging or worsening psychiatric symptoms lasting for over 72 h after psychedelic use, a small number of survey responders were contacted and offered an interview. We aimed to conduct interviews with a total of 15 individuals—after which, recruitment for interviews would be stopped in order to focus on the analysis stage of this study.

Priority for offering an interview was defined as follows:

1. Person reports being diagnosed with a psychiatric disorder including psychotic features (e.g., schizophrenia, bipolar, etc.) *after* a psychedelic drug experience.
2. Person reports emerging or worsening other psychiatric symptoms *after* a psychedelic drug experience, defined in the survey as: symptoms of depression or anxiety, abnormally elevated mood, extreme and problematic distractibility, impulsive behaviour, psychological distress, intrusive thoughts, anxiety, panic, sleep disturbance, paranoia, delusional thinking, auditory hallucinations such as hearing voices, complete loss of pleasure, obsessive thoughts or behaviours, addictive thoughts or behaviours, self-harm, suicidal thinking, planning or behaviour.
3. Person reports symptoms of HPPD emerging *after* a psychedelic drug experience. HPPD was defined according to Diagnostic Statistical Manual (DSM) criteria as: any of the following that cause significant distress: halos or auras surrounding objects, trails following objects in motion, difficulty distinguishing between colours, apparent shifts in the hue of a given item, the illusion of movement in a static setting, air assuming a grainy or textured quality (visual snow or static), distortions in the dimensions of a perceived object, and/or a heightened awareness of floaters.

Interviews were semi-structured, up to 90 min in length, and led by two study researchers, of which one was a mental health professional (NK or PS) with at least 3 years’ clinical experience.

### Inclusion and exclusion criteria

The main inclusion criteria for the present study were: Having experienced at least one of the symptoms listed above, lasting for at least 72 h after a psychedelic experience—thought by the participant to be caused entirely, mostly, or, at least in part, by the psychedelic they took. Psychedelic was here defined as predominantly serotonergically-acting psychedelics, namely LSD, psilocybin/magic mushrooms/truffles, DMT, ayahuasca, 5-MeO-DMT, mescaline (synthetic or plant derived), and the psychedelic-like drug MDMA/ecstasy. Participants were required to be at least 18 years old, able to communicate in written and spoken English, having access to the internet and an email address, and willing to participate in 1–2 interviews, if offered.

Exclusion criteria were defined as: (1) person used a psychedelic within two weeks of the interview, or (2) person lacks the capacity to undergo the interview phase of the study, e.g., if currently floridly psychotic. This was assessed by a clinical interviewer at the beginning of the video interview. The capacity assessment was done according to the Mental Capacity Act 2005^44^.

### Analysis

Data collected through the online questionnaire was structured and descriptive analyses were calculated in Excel, to be presented in tables and diagrams. Interviews were transcribed and summarized into brief individual case reports. Additionally, thematic analysis was used to find themes and patterns between the fifteen interviewed participants. A hybrid of inductive and deductive thematic analysis was used, where the inductive approach involves allowing the data to determine the themes, and deductive includes pre-existing information that we were actively looking for in the data^43^. The latter approach included, for example, inferences based on the findings of prior research as well as anecdotal reports of adverse responses to psychedelics.

### Ethical considerations

This study was approved by Imperial College Research Ethics Committee (ICREC)—reference number 21IC7184. The study was performed in line with the principles of the Declaration of Helsinki^74^.

To ensure access to mental health support throughout the study, given the sensitive nature of this study’s focus, we made available, to each participant, a link to a mental health support organisation such as a free volunteer counselling charity (e.g., the Samaritans, UK). Moreover, all interviews were done with the presence of an experienced mental health professional.

Participants were required to give informed consent prior to each of the two phases of the study. After completing the questionnaire, participants were invited to submit an email address or phone number and express their consent to being contacted about participation in a subsequent video or phone call interview. At the beginning of, and throughout the interview, the mental health professional present carried out a capacity assessment, accordingly to the Mental Capacity Act 2005^44^.

To protect the identities of our participants, each individual was allocated an individual participant ID number on enrolment in the study. Interviews were audio recorded for transcription purposes, and deleted after transcription. Personally identifiable information was deleted in the transcription process and the transcripts were pseudonymised.

## Results

### Phase 1: questionnaire

#### Participant flow and attrition

**Figure 1:**
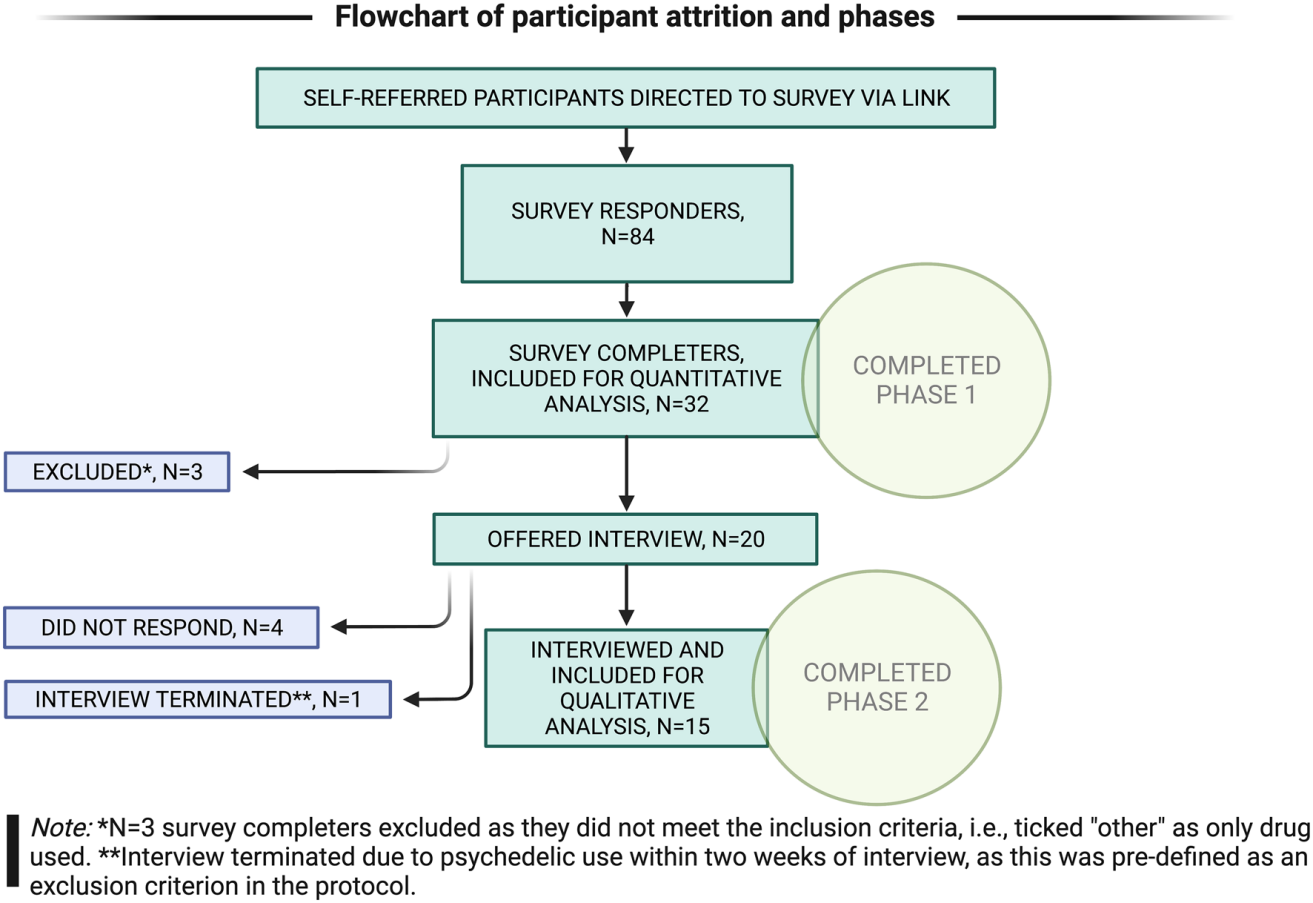
Flowchart of participant attrition and phases.

Figure 1 the above flowchart shows participant attrition during the two study phases. 84 participants completed consent but only 32 of these completed the whole survey. The complete survey responses were reviewed. The total number of interviews was pre-decided to be 15. Of the 20 contacted participants, four did not respond, and one was excluded as this person did not meet the inclusion criteria for this study (i.e., had consumed a psychedelic within two weeks before the interview). The latter was offered rescheduling to a later interview but declined. The total number of survey completers was 32 (Table 1).

**Table 1:** Demographic profile of survey completers, including distribution of specific drug-use (n = 32).

	N	%
Gender		
Female	13	41
Non-binary	1	3
Male	18	56
Geographic location*		
United States	8	25
United Kingdom	6	19
Germany	3	9
Canada	2	6
Other	11	34
No answer	2	6
*Note: Specific countries reported by n = 2 or more listed, the remaining included in ‘other’		
Past or current mental health diagnosis**		
None	10	31
Yes	22*	69
**Note: Of which 13 were diagnosed after the experience		
Diagnosed before the experience (n = 9)***		
Depression	4	44
Anxiety	1	11
ADHD	1	11
Bipolar disorder	1	11
Personality disorder	2	22
PTSD	2	22
Eating disorder	1	11
Dissociative disorder	1	11
***Note: Those with a psychiatric diagnosis were able to tick more than one option, hence the total number larger than number of diagnosed participants		
Drugs used		
LSD	16	50
Psilocybin	11	34
MDMA	11	34
DMT	5	16
Ayahuasca	3	9
Other****	4	13
****Note: The category ‘other’ included cannabis, risperidone, *[prescribed psychotropic medication], and undefined drugs (i.e., participants ticked “other” without further explanation).*		
Use patterns		
Used only one psychedelic	22	69
Polysubstance use	10	31

Across the sample of 32 survey completers, 30 completed the Challenging Experience Questionnaire (CEQ) and reported a total mean score of 62.1 ± 31.8—which is numerically higher than previously reported average scores; e.g., 19.7 ± 16.4 in a large-scale (n = 379) prospective psychedelic survey study by Haijen et al.^25^, and 33.3 ± 22.7 in a sample of individuals (n = 886) who, via their own initiative, participated in one or more psychedelic ceremonies, i.e., Kettner et al.^11^. Our sample were also remarkably higher across the seven dimensions of the CEQ, comparison showed in Table 2.

**Table 2:** Comparison of CEQ scores across studies.

	Total	Death	Fear	Grief	Insanity	Isolation	Paranoia	Physical distress
This study	62.1 ± 31.8	4.2 ± 4.3	18.2 ± 8.0	15.3 ± 9.8	8.1 ± 6.0	6.8 ± 6.2	1.3 ± 2.4	8.3 ± 6.0
^11^	33.3 ± 22.7	1.5 ± 2.7	6.26 ± 6.4	12.1 ± 8.2	2.46 ± 3.48	3.83 ± 4.08	1.02 ± 1.68	6.26 ± 5.18
^25^	19.7 ± 16.4	9.66 ± 21.4	21.8 ± 23.4	22.3 ± 22.8	18.03 ± 23.5	21.4 ± 24.6	8.34 ± 15.2	23.1 ± 18.6

**Figure 2:**
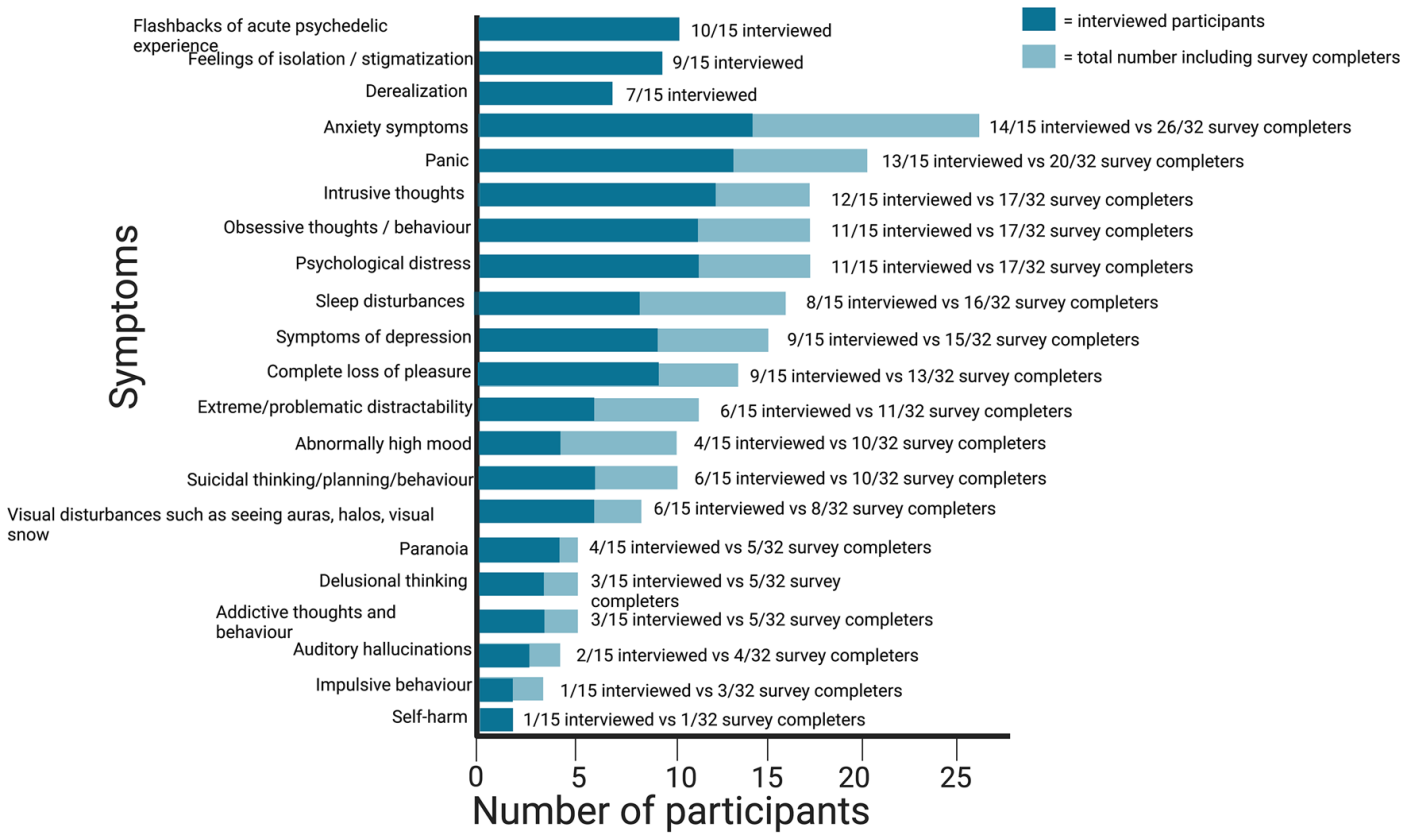
Symptoms reported by 32 survey completers and 15 interviewed participants.

Figure 2 the darker color of each staple show prevalence in the interviewed sample, and the lighter color show prevalence in survey completers. For example, 14 of 15 interviewed participants reported anxiety symptoms, and 26 of 32 survey completers reported anxiety symptoms. The three symptoms at the top of the figure were found in interviews and not listed in survey, thus only interviewed participants reporting them (see page 8 for a description of these). The most common emergent symptom type in our sample of 32, was anxiety (26 of 32 participants, 87%), shortly followed by panic (20 of 32 participants, 63%), see Fig. 2 below.

### Phase 2: interview phase

#### Summary of interviewed participants as a group

Fifteen participants completed the full survey and participated in a semi-structured interview. Of these, 8 were female, 1 nonbinary/third gender, and the remaining 6 were male. Mean age at time of the psychedelic experience was 25 (SD = 7.4), and the time-since-the-experience ranged from 2 months to 25 years (M = 6.8 years, SD = 8.5). Some participants considered themselves fully or mostly recovered when we spoke to them, but for most there was some ambivalence about the experience and whether or not the symptoms linked to it were still remaining or impacting their lives (more detailed information about this can be found in supplementary information, supplementary note 2, page, 6). LSD was the most commonly used drug (reported by 7 of 15), followed by MDMA (6 of 15) and then psilocybin (4 of 15). Eleven interviewed participants reported exclusive use of one drug during their experience, leaving n = 4 cases of acute polydrug use.

Case-by-case summaries of all 15 interviewed participants can be found in supplementary information, page 1.

#### Qualitative analyses

##### The nature of acute experience

As the high CEQ rates across the full sample of 32 may have indicated, a majority of our interviewed participants (11 of the 15 interviewed, i.e., P1, P3, P4, P10, P12, P15, P20, P22, P24, P26, P28) described their acute psychedelic experience as negative and/or frightening (e.g., as a ‘bad trip’). All of these 11 individuals had taken a classic psychedelic as defined above. The remaining four, of which three had used MDMA/ecstasy (plus possibly other stimulants), and one LSD, reported generally pleasant and positive acute experiences.

##### Symptom profile of interviewed participants

To get an overview of the nature of participants’ negative responses, 18 symptoms were listed in the phase 1 survey (see Fig. 2 for full list of symptoms listed in survey and number of participants reporting each one)—anxiety symptoms (93%) and panic (87%) were the most prevalent in the interviewed sample. In addition to this list, three more non-listed symptoms were described by seven or more interviewed participants and therefore we retrospectively added them to our list of symptoms. We categorized these emerging symptoms as ‘*flashbacks of acute psychedelic experience*’*,* ‘*derealization*’, ‘*disconnection (including sense of stigmatization)* ’ and ‘*flashbacks of acute psychedelic experience*’. These are described below:

*Derealization:* 7 of the 15 interviewed participants (P3, P4, P10, P20, P22, P23, P28) i.e., 47% spoke of derealization. This was described, for example, as “having daily out-of-body-experiences” (P20), “reality felt thin or unstable” (P3), “[being] completely disconnected from the universe” (P10), and P23 said: “It was a… derealization… […] like, there was something wrong with my normal world?” (P23).

*Disconnection (including sense of stigmatization):* 7 of 15 (P1, P3, P4, P10, P15, P25, P26), i.e., 47% described feeling isolated with their mental health problems following their psychedelic experience. For example, one person described: “…this fundamental sense of aloneness, like no one can help me. I´m slipping into hell, I’m… my soul is alone, I’m completely disconnected from the universe” (P10), and “I couldn’t talk to people about it because then you’re just crazy” (P4). This sense of isolation and critical judgement was described as anxiety-provoking and, by some, as worse than the actual symptoms: “But it’s the kind of vilification of HPPD, and for me, it’s not so much about having the symptoms, it’s the isolation.” (P26). Difficulties in asking for professional help, due to the stigma related to psychedelics, was also mentioned by three participants (P25, P3, P15).

*Flashbacks of acute psychedelic experience:* 12 of 15 (P1, P3, P4, P10, P12, P15, P20, P22, P23, P24, P26 P28), i.e., 80%, reported that the prolonged adverse responses felt very similar and/or connected with an unpleasant (e.g., frightening) psychedelic experience. Some described this connection with the term ‘flashbacks’ or ‘emotional flashbacks’—implying a PTSD-like intrusive psychological reoccurrence of experienced ‘trauma’. For some individuals, this perpetual psychological struggle linked to the actual drug experience, was still ongoing when they participated in the study. Eleven of these 12 participants had used a classic psychedelic and described the acute experience as overtly negative and/or frightening.

In one anomalous case, experiencing anxiety-provoking flashbacks of the psychedelic experience was talked about by participant 23 who had used MDMA and described her acute experience as pleasant and positive:

“So, maybe it’s important that at this point I was hearing the same music again as I had when I took the MDMA. And it still felt the same, like very, very deep listening to the music. And then suddenly, I had this great anxiety. It was from one second to another. It was so overwhelming, and yeah, it didn’t stop for two or three days.” (P23).

Some reported these ‘flashbacks’ being triggered by cues that reminded them of the psychedelic experience, and for others, memories could arise without an obvious trigger (e.g., P28). The onset of these symptoms occurred directly after the psychedelic experience for e.g., P28, although for other participants, the symptoms had started 2 weeks (P15) or 2 months (P3) after the psychedelic experience.

Moreover, one (P25) of the 11 participants who had used a classic psychedelic and described a positive acute experience on LSD reported symptoms of HPPD, such as visual disturbances, as the main prolonged adverse response, and anxiety as a secondary symptom caused by the HPPD.

The theme of an association between an overtly challenging acute experience and subsequent prolonged adverse psychological responses was, however, otherwise very reliable. For example, one participant (P22) described how during the psychedelic experience, she saw traumatic events, including violence and rape, that she had personally experienced in her own life, but she saw it from an externalized perspective (i.e., she saw herself but was not in her body). She described the experience as extremely frightening, and although already knowing that these things had happened, seeing it all from outside compounded the significance of the event. She described this as overwhelming, and reported experiencing extreme anxiety, flashbacks, nightmares and what she perceived to be agoraphobia, for many years afterwards. This individual was interviewed around 12 years after the drug experience, and while most symptoms had ceased, she said that the experience had changed her in many respects. Thus, here the effect seems to be one of re-traumatization or the recovery of traumatic material that was not psychotherapeutically processed.

##### Risk factors

Thematic analysis led to the identification of four potential risk factors, with several subthemes within each. These are as shown in Table 3 below.

**Table 3:** Themes of potential risk factors.

	Theme	Number of endorsing participants
A.	**Drugs and patterns of use**	13
	A.1. Unknown or unusually high quantities	10
	A.2. Frequent use prior to the event	4
	A.3. Questionable drug purity or quality	3
	A.4. Polysubstance use (including prescribed medications) and/or discontinuing medication abruptly	5
B.	**Personal- or family history of psychiatric disorders and mental health issues**	12
	B.1. Personal history of psychiatric diagnosis *prior* to the experience*	4
	B.2. Personal history of undiagnosed mental health problems	10
	B.3. Post experience formal diagnosis	5
	B.4. Family history of psychiatric diagnosis	2
	B.5. Family history of undiagnosed but suspected mental health problems	8
C.	**Negative or unsafe expectations / environment**	12
	C.1. Unsafe environment or stressful incidents happening during the experience	6
	C.2. Negative priming	4
	C.3. Stressful time or major life changes surrounding trip	9
D.	**Problems in interpersonal relations**	8
	D.1. Relationship tensions with those present for experience	6
	D.2. Lack of social support system during or after experience	5

*Note: Five participants reported experiencing the symptoms prior to the experience but receiving a formal diagnosis after.

A. ***Drugs and patterns of use***
A.1. ***Unknown or unusually high quantities***

Of course, unknown quantities and purity is an almost unavoidable consequence of the illegal status of and associated lack of regulatory quality-control on psychedelics—and therefore represents a risk created by drug policy. As with all things consumed, higher dosages are likely to increase the risk of adverse events and such dose dependency for adverse events is true for psychedelics—with different risks becoming more likely with different substances e.g., psychological risks with classic psychedelics^23,26,73^ and toxicity risks with higher doses of MDMA^45,46^.

A majority (10 of 15) of interviewed participants were either uncertain about what dose they had taken or reported having taken a dose that was unusually large compared to what they had taken before. One participant (P24), who, earlier in the interview, had described himself as being very sensitive to LSD, said the following about his dosing at the event that he thought had led to the prolonged adverse psychological responses:

“I had gotten this vial [of LSD] from a friend of mine and was really excited to try it […] had woken up at like two in the morning [after only 2–3 h of sleep] because I was so excited and I couldn’t go back to sleep […] started taking one drop from this vial every hour, from 2 until probably 7 or 8 am […] It could total up to like 500 or more, which is a very high dose for LSD.” (P24).

A.2. ***Very frequent use prior to the event***

Four participants (P4, P7, P24, P25) reported very frequent use during the months or years prior to the event they thought had triggered their adverse responses. For example, “it´s not only that I took it that day, I had also taken it the day and week before.” (P25), and “I kept taking these substances over and over, and then it kind of got to this point where I really couldn´t anymore, because it would just be so negative, It would be very adverse and I started having panic attacks.” (P24).

A.3. ***Questionable drug purity or quality***

The quality of the drugs was not always known to the participants. P20 reported being dosed against her will—thus not knowing anything about the drugs until later comparing the phenomenology of her experience with other’s reports and concluding that it was likely to be DMT that she received. P24 received their LSD from a questionable source, i.e., a friend who made it himself. However, as stated earlier, such scenarios are likely to be the norm with illegal use of psychedelics due to a lack of regulatory quality-control. A hindsight bias—and a deflecting to an explanation of convenience—could also contribute to a laying blame on drug quality when this was not actually a key contributing factor.

A.4. ***Polysubstance use (including prescribed medications) and/or discontinuing medication abruptly***

Four interviewed participants (P7, P15, P22, P28) 26.7% had used a psychedelic in combination with other psychedelic- or non-psychedelic drugs, of which one (P28) also in combination with prescribed Risperidone (i.e., antipsychotic medication). Another participant (P1) had, shortly before the experience, decided to discontinue his prescribed ADHD medication. See Fig. S1 in supplementary information for further information on which drugs were used together. In addition, 10 (31%) of the 32 survey completers reported mixing of drugs.

B. ***Personal- or family history of psychiatric disorders and mental health issues***

##### Personal mental health

1. ***Personal history of diagnosed psychiatric disorders prior to the experience***

Four of 15 (27%) interviewed participants reported psychiatric diagnoses prior to the experience. These includes Attention Deficit Hyperactive Disoder (ADHD) and Major Depressive Disorder (MDD) (P1), MDD in the past, although recovered (P12), severe depression with psychotic features (P28), and bulimia (P23).

B.2. ***Personal history of undiagnosed mental health problems prior to the experience***

Another six participants reported struggling with a variety of (undiagnosed) mental health issues prior, including undiagnosed seasonal affective disorder and “pretty standard occasional anxiety” (P10), eating disorder (P12), trauma, instability in family and “bad mental health” (P22), visual snow (P25), anxiety (P26), and taking antidepressant medication in the past (P12). Hence, most of the interviewed participants (i.e., 10 of 15, 67%) reported some form of mental health problem—diagnosed and undiagnosed.

B.3. ***Post experience formal diagnoses***

Five participants talked about having experienced mental health difficulties before their psychedelic experience, and claimed these symptoms were exacerbated and formally diagnosed after the experience: Two participants (P4, P24) were diagnosed with bipolar disorder shortly after the psychedelic experience; both had a family history of bipolar and believed they had pre-existing attenuated symptoms prior to the experience. Other diagnoses received after the experience (although participants reported symptom being present prior) included depression (P7, P15, P23), anxiety (P15, P23), borderline and PTSD (P7), binge eating disorder (P1) and PTSD with psychotic features (P20).

No one reported entirely new psychiatric disorders emerge after the experience that were essentially non-existent before, i.e., even P25 reported visual snow prior to his high-dose (400mcg) LSD experience.

##### Mental health in immediate family

B.4. ***Family history of diagnosed psychiatric disorders***

Three participants (P4, P7 and P24) talked about their family members being diagnosed with bipolar disorder—as mentioned above, one of these individuals went on to have their own diagnosis of bipolar disorder shortly after his psychedelic experience (P24).

B.5. ***Family history of undiagnosed mental health problems***

Eight of the 15 (53%) participants talked about having family members with undiagnosed mental health problems.

Taken together, only two (i.e., 13%) of the 15 interviewed individuals reported no personal or family history of either diagnosed or undiagnosed mental health problems; thus, by deduction, 87% of the interviewed sample had personal or family histories of psychiatric illness.

C. ***Negative or unsafe expectations/environment***
C.1. ***Unsafe environment or stressful incidents happening during the experience***

Some participants talked about being in an environment which they did not feel safe in, e.g., with a group of friends they did not know well or feel fully comfortable with (P1 and P22). Others talked about incidents happening during the experience, which pivoted the psychedelic experience in a negative direction. The latter was, for example, when someone they were sharing the experience with started struggling (P26 and P28), as well as being kicked out of someone’s parents’ house that they were in, at the timepoint when the drug effects were beginning to intensify (P28 again).

One participant reported an overall pleasant experience on LSD until she happened to see her own face in the mirror:

“…and what I saw was just… not OK, I can’t really explain it, but it’s kind of… I looked like some kind of evil witch, evil thing, I don’t know, it just kind of shattered my sanity just looking at it […] I was pretty inexperienced, I didn’t know that you’re supposed to accept things and so I was basically trying not to have a bad trip. […] I had no foothold on sanity, I couldn’t grasp onto anything. Nothing I was experiencing or perceiving seemed stable. […] Like, before I looked in the mirror, I remember thinking that I’d heard you’re not supposed to look in the mirror, and for some reason I just decided to do it anyway, so I think maybe I had a negative expectation in my head.” (P3)

Another participant (P20) was dosed against her will and then sexually abused during the experience. She was diagnosed with PTSD with psychotic features after—and as a result of—the experience, and said the following about it:

“I was just, like, smoking a joint with my friend […] It was really frightening, I had no idea what was going on. And then he had me, he was like “repeat after me, say `I am God` […], and I was like “you are God”. […] And so, like at that point I was like incoherent, but I wasn’t having extreme visual effects, it was more… vibrational. And then when I closed my eyes it was kind of my entire body was just like vibrating. […] It was terror. It was absolutely terrible. Uh, it felt like I was dying and like I was speaking to this figure and this figure basically was… showing me my entire life and I experienced life review where my life just flashed before my eyes. And what was happening was that I was being sexually assaulted, it was absolutely terrible. And then I woke up. It was kind of like, it was happening, and then when it ended, I was sober, and I was lucid.” (P20)

C.2. ***Negative priming***

Some participants cited apprehensive or negative expectations as having been a contributing causal factor. For example, P23 described a prior negative bias against psychedelics, but decided to try it in an attempt to understand her boyfriend better, and P10 described her experience being negatively primed by a difficult Ayahuasca ceremony the night before:

“My thought is that kind of primed it… like, there was something [physical] that was overwhelming [i.e., referring to the ceremony the night before] that I think also led to the second night being just complete hell. […] Yeah. I think I went in with a little fear.” (P10)

C.3. ***Stressful time or major life changes surrounding trip***

Seven of the 15 (47%) participants reported experiencing significant life stress around the time of their experience, as well as stressful and/or traumatic events transpiring during it. Examples ranged from: stress or uncertainty in their professional lives (P12, P15, P3), currently working through difficult past experiences in therapy (P12), not feeling well that day (P24), uncertainty in romantic relationships (P16, P23), a recent (P22) or unprocessed (P15) breakup.

D. ***Problems in interpersonal relations***
D.1. ***Relationship tensions with those present for experience***

Five of 15 (33%) participants described taking the psychedelic with or in the presence of someone with whom there was relationship tensions. For example, P15, had been with someone that owed them a lot of money. He believed that the tension between them and the anger he felt towards this person was a significant factor causing the negative outcome. Other participants talked about taking the psychedelic with the intention of working through relationship issues with their romantic partner e.g., “to get through a rough patch in the relationship” (P3), as well as ‘tripping’ with a partner that they did not feel fully comfortable with:

“I had some afterthought about the relationship at the time and I wasn’t feeling, like, fully comfortable with him and just the feeling that I can’t communicate with him, and I can’t be with him in the experience was freaking me out also on this interpersonal romantic level.” (P12).

Two other participants described a negative transfer caused by someone else in their group starting to struggle on a psychedelic (P26, P28).

D.2. ***Lack of social support system during or after experience***

A lack of social support during or after the experience was described as a potential contributing factor by five of the 15 (33%) participants. For example, some reported not feeling able to call anyone when the psychedelic experience became frightening. This participant had not told anyone that she would take a psychedelic (P12). Another participant reported reaching out to therapists who appeared to have a negative bias towards drugs (P15). Others reported encountering health care professionals who did not have competence with psychedelic drug related symptoms, e.g., HPPD (P25 and P26).

## Discussion

The present study sought to identify and examine long-term negative psychological responses to psychedelic drugs, where ‘long-term’ was defined as lasting longer than 72 h after the experience and ‘psychedelic’ was defined as a range of classic psychedelics plus MDMA. We used a two-phase approach involving an initial onboarding questionnaire designed to collect quantitative descriptive data and screen for a subsequent interview phase; the interview phase yielded richer qualitative data and constitutes the core data for this study.

The main motivation for this study was to address a dearth of research on long-term negative psychological responses to psychedelics. We sought to glean some insight into why such responses occur, with the aim of informing on risk mitigation messaging and strategies. Self-selective volunteering and recruitment plus confirmation biases may have created a skew in previous research findings on psychedelics that may have inflated positive and downplayed negative responses. This may be a particular issue in online surveying [e.g.,^15,25^] where there is minimal control on the recruitment process and advocates of psychedelic medicine may feel more inclined to engage. Our solution to this challenging issue has been to create a new study focused exclusively on negative psychological responses. It was hoped that this selectively (negative) focus would appeal to individuals who have experienced such responses—as they would feel motivated to share their experiences when otherwise they might feel disinclined to engage or even neglected.

### The difficult question of prevalence

Due to our selective recruitment approach and the retrospective study design, we strongly caution against drawing inferences on the prevalence of negative psychological responses to psychedelics from the present study’s findings. Population studies^12,47^, multi-site trials [e.g.,^48^] and meta-analyses of controlled studies^49^ would be better suited for this purpose. Dubious case reports could be used as opportunism for over-stating the prevalence of harms of psychedelics—as we fear has happened recently^50^.

Eighty-four questionnaires were submitted in the present study but only 32 were completed in full, for an attrition rate of 62%. The questionnaire’s length (average completion time = 30 min) may have contributed to these rates. Sixty-two percent attrition rates are broadly consistent with drop-out rates at 2 weeks post use in prospective surveys we have previously conducted, where young age was the strongest predictor of drop-out ^16^. The questionnaire was open for no longer than 6 months, a relatively brief period. We had hoped for more responses—and encourage a longer-recruitment period and shorter onboarding questionnaire if similar studies that are to be carried out in the future. The questionnaire was shared on fora dedicated to certain negative response types (e.g., a HPPD forum of Reddit) as well as by popular figures with large audiences on social media—such as Michael Pollan and Tim Ferriss. Biased sampling could therefore have occurred in either direction i.e., for or against psychedelics. Future studies, ideally with larger samples and better recording of sources of recruitment, could seek to examine the impact of recruitment sources on outcomes.

As stated above, our study does not allow for inferences to be made on the prevalence of negative responses to psychedelics in a representative and large population (such as psychedelic users in the United States), but we do feel we can comment on the prevalence of specific psychiatric symptoms reported by those *within* the small sample of 32 questionnaire completers and the even smaller sample of 15 individuals who were interviewed. Within both of these small samples, anxiety was the most prevalent symptom type described, reported by 81–93%. This compares with lower rates for overtly psychotic (e.g., highly unusual/magical ideas or auditory hallucinations) or HPPD-specific symptoms, which were reported in the questionnaire sample by 13–16% (psychotic symptoms) and 25% (HPPD symptoms), respectively, and 13–20% (psychotic symptoms) and 40% (HPPD symptoms) in the interviewed sample of 15. However, despite relatively low rates of overtly psychotic symptoms and diagnoses, psychotic-like symptoms were not uncommon in the interviewed sample, e.g., 47% (i.e., seven of 15) described derealization, or ‘losing connection with reality’ after the experience itself.

Defining HPPD symptoms presents another challenge. The DSM-5 require at least one of nine symptoms (as defined on page 4 and here:^40^) to be present after psychedelic use for a diagnosis of HPPD to be valid. However, importantly, the symptoms must “cause [the affected individual] clinically significant distress or impairment in important areas of functioning, such as social and occupational environments”^40^. Only one of the 32 survey completers reported a formal diagnosis of HPPD, but a larger proportion described some symptoms (6–19%). Among the 15 interviewed participants, four described experiencing or having experienced what they referred to as HPPD-like effects. Of these four, only two found the effects distressing and negatively impairing (the latter two were both recruited through a reddit forum focused on HPPD), but none of them had a formal diagnosis of HPPD. Relatively high prevalence of occasional HPPD symptoms, but very low prevalence of HPPD diagnosis and perceived negative impact of the experienced symptoms, is also consistent with the findings from previous studies^51^ including a recent one by our group, where 68 of 212 respondents (32%) reported at least one HPPD symptom. However, only one of these 68 (i.e., 3% of those reporting any HPPD symptom) experienced the symptoms as distressing^59^.

Rates of formal diagnoses of psychotic disorders arising after psychedelic-use were low (i.e., three of 15 or 20%). Specifically, in the interviewed sample, there were two new cases of bipolar diagnoses, and one case of PTSD with psychotic features. Other cases in the questionnaire sample of 32, included one new case of schizoaffective disorder, one further case of bipolar disorder (i.e., three in total), two cases of borderline personality disorder, two cases of PTSD, and one case of HPPD. In the sample of 32, most new diagnoses arising after psychedelic-use were depressive or anxiety disorders (i.e., six cases in total).

It is difficult to draw inferences on the prevalence of formal diagnoses emerging after psychedelic-use in a broader population or the prevalence of specific diagnoses. Anxiety and depression are the most prevalent psychiatric symptom types. Given the historic spotlighting of enduring psychotic symptoms^52,53^ and HPPD^54^ after psychedelic-use, we felt particularly motivated to discover cases of these phenomena in the present study. We recognise, however, that behavioural and cognitive disorganisation linked to psychotic symptomatology may make it less likely that individuals suffering from these symptoms would volunteer for our study (as well as previous survey studies)—or indeed persist with the process through to interview. Thus, again, we are left with the difficult scenario of not knowing whether cases of enduring psychotic symptoms after psychedelic-use are extremely rare—as some have suggested^12^—or whether our methods for detecting such cases remain flawed.

Future studies could be carried out that focus on cases of specific symptomatology or diagnoses allegedly arising after psychedelic use—such as those of a psychotic type. Additional methodology, such as next-of-kin and/or carer interviewing, could be used to overcome inadvertently exclusory recruitment approaches—and gather other independent perspectives on the same individual cases. Future research might also better examine individuals’ motivations for volunteering and their intentions for the psychedelic use, as well as clearer information on age at the relevant time of use as well as set, setting and what has been called psychosocial ‘matrix’ (1997). New studies might also examine long-term physical symptoms attributed to psychedelic use, acute as well as long-term negative responses, and include drugs that were excluded from our study—such as ketamine and cannabis. One previous study of ours did find a link between co-use high potency cannabis and challenging psychological experiences under psychedelics (Kuc et al.^75^).

### The difficult question of causality

Acknowledging that our methodology cannot enable us to draw inferences on the prevalence of long-term negative psychological responses to psychedelics in a broader population than the small one studied here, we designed this study in such a way that might enable us to speculate on the causes of iatrogenic outcomes. The two-phase approach was conceived with this aim in mind e.g., with phase 1 collecting quantitative data on certain set and setting factors—plus basic demographics and use parameters, and phase 2 collecting a richer qualitative perspective on each case via interviews. It was via the interviews that we felt we may be able to glean insight on causality.

Distilling the information gathered from this approach, we felt we were able to identify some consistent themes across the sample pertaining to the question of causality. These variables can be categorised into 3 major domains: namely, (1) factors pertaining to the individual’s psychological vulnerability (including their young age), (2) (negative) set, setting and/or matrix factors, and (3) factors linked to the substance itself—and most commonly, its (excessively high) dose and particular action. It is worth noting that these factors are entirely consistent with those highlighted in previous work in relation to the hypothesised context-dependency of responses to psychedelics [e.g., see Fig. 2 in^30^].

These factors have been unpacked in some detail in the results section and so we refrain from repeating this process here. Instead, we will discuss a parsimonious mechanistic model in which these factors interact. This model highlights the pro-plasticity effect of psychedelics, where plasticity is defined in its broadest and simplest sense as the ability of something to be shaped or moulded. Thus, an increase in plasticity implies that the object in question (e.g., the mind, brain or behaviour) is more easily shaped or moulded. If we acknowledge that increasing plasticity is a basic and direct action of psychedelics, then simple logic can be used to explain how increasing the dosage of the drug will increase the impact of any contextual factors on the individual’s response, and if these factors are negative, a long-term negative psychological response will be more likely. Related ideas have been discussed previously^55,56^.

It is not trivial to extricate individual vulnerability from ‘set and setting’. For example, the variable ‘set’—refers to psychological factors an individual brings to the experience, such as their expectations and mood prior to dosing; however, such factors are difficult to distinguish from: (1) current life stress and (2) the trait-level character of the individual, which will have depended, in no small part, on their life experiences in development^57^ and adulthood. One could attempt to simplify the ‘individual vulnerability’ factor by referring to an individual’s biologically based disposition (e.g., as influenced by genetic factors) but even this cannot be extricated from environmental influence [e.g.,^58^]. Moreover, according to this study’s data and currently unpublished findings^59^ we would argue that young age should be included as a vulnerability factor.

Thus, we are left with an intentionally parsimonious model that emphasizes a key axis of plasticity that is activated dose-dependently by the psychedelic and may involve some individual differences in sensitivity—interacting with a critical second dimension, which refers to the context in which the use occurs. Here, ‘context’ can be used as an umbrella term to subsume the sub-dimensions of set, setting and matrix [e.g., as was done here^30^]. Individual sensitivities could be subsumed into both dimensions, where e.g., greater sensitivity to psychedelics would positively interact with their direct pro-plasticity effects but could also be regarded as a ‘negative context’—e.g., in terms of adverse life experiences or psychological vulnerabilities brought to the experience. Literature on differential susceptibility is relevant here^60^. Thus, according to our simple model, a long-term negative psychological response to a psychedelic will depend on activating plasticity in interaction with a sub-optimal context. This simple model is entirely consistent with classical perspectives on psychedelics that have emphasised the importance of ‘set and setting’^29^, ‘extrapharmacological’^31^ or ‘contextual’ determinants of outcomes^30^ as well as the description of psychedelics as ‘nonspecific amplifiers’ (i.e., of psychological phenomena or states)^61^.

Of the 15 participants interviewed, most, if not all, fitted this basic model. The model feels especially compelling in relation to those (12/15) cases where a classic psychedelic had been taken, and in all but one of these cases, the long-term sequalae appeared to follow an initial challenging experience or ‘bad trip’. In several of these cases, a negative set and setting going into or during the experience appeared responsible for a subsequent challenging acute experience that may or may not have been compounded by a negative psychosocial ‘matrix’^32^ before, during or after the experience. In at least one of these cases, the set and setting was overtly traumatic, involving alleged surreptitious dosing and sexual abuse. The victim of this abuse was diagnosed with PTSD with psychotic features afterwards, plus borderline personality disorder. Sexual abuse and malpractice in relation to psychedelic-use has recently been covered in journalistic work^62^ but has, to our knowledge, had minimal coverage in scientific press^63^. The re-traumatising potential of psychedelic experiences is arguably better covered, however^22,64^, and is a risk, particular in uncontrolled, unregulated and/or non-therapeutic settings. See^55^ for a relevant discussion.

Four of the 15 (27%) cases did not, however, fit the basic 2-factor psychedelic response model quite as neatly. Notably, 3 of these cases involved MDMA-use preceding sub-acute low mood or depression (P7, P16, and P23) and one involved LSD-linked HPPD symptoms (P25). It was difficult to ascertain whether individual vulnerabilities and/or a negative psychosocial matrix had contributed to the post-MDMA symptomatology or whether the specific action of this particular compound e.g., causing post-acute changes in serotonin metabolism and its availability for transmission^65^ had also played some role. In the post-LSD HPPD case, the dose was high (400 mcg) and the person was of young age (aged 21), and it seems plausible that the symptoms of HPPD had triggered or worsened their general psychological presentation i.e., causing distress, perhaps compounded by prior psychological vulnerabilities.

When viewed in this way, one could argue that all twelve of the cases involving a classic psychedelic, can be explained by the two-factor model of plasticity × context. Moreover, one could also argue that, pending adherence to protocol, none of the (15 interviewed) cases could have occurred in a clinical trial scenario, i.e., some individuals would have been excluded from trials and dosage and context would have been properly managed. This argument deserves careful consideration as it has implications for drug policy. More specifically, the cost–benefit evidence derived from psychedelic therapy trials cannot be used to infer the cost–benefit profile for psychedelic-use in a scenario of unregulated legalization.

Ours is not the only study to have specifically assessed negative responses to psychedelics [see, for example,^21,23,50,66,70^; however, to our knowledge, it is the only one to have selectively invited individuals who believed they had suffered long-term negative psychological responses to take part in a two-phase study culminating in a semi-structured interview. In addition to poor set and setting factors^25,71^, previous work has identified young age, drug mixing^66^, and certain personality^67^ and vulnerability factors^66^ as predictors of negative psychological responses, and a separate study of ours using prospective surveying found that a personal history of personality disorder diagnoses conferred special risk for negative long-term psychological responses to psychedelics^34^. These risk factors could be more easily monitored and safeguard against with better psychedelic drug education and regulation—and this would arguably be easier to achieve with responsible changes in drug policy^68^ e.g., via legal regulated psychedelic therapy^69,72^, as well as better access to existing risk mitigation services (e.g., https://firesideproject.org).

Finally, we acknowledge that the present study’s data derives from participant testimony. We cannot confirm the purity, potency and dosages of drugs used or the accuracy of other information provided to us by the respondents. Some of the relevant experiences had occurred up to twenty-five years prior to engagement in our study, and the mean time since the experience was 7 years. Thus, the information relayed to us is vulnerable to recall inaccuracies.

## Conclusions

In conclusion, prolonged adverse psychological responses to psychedelics are difficult to study but it is essential that we endeavor to do so. Researching vulnerable populations is fraught with challenges but in the present case, the apparent low prevalence and sensitivity of the focal phenomena combined with participant engagement issues, compound the challenge. Here, we used a mixed methods and selective recruitment approach in an attempt to overcome these challenges. Our process approach yielded insight on possible causal factors contributing to the adverse events and inspired a simple model intended to highlight the essential context dependency of most—if not all—cases of prolonged negative psychological responses to psychedelics. We hope this small, proof-of-principle study will inspire others to advance on our methods to deepen our data pool of such important cases so that their occurrence can be better understood, and likelihood, minimized.

### Supplementary Information

Supplementary Information 1.

Supplementary Information 2.

## Data Availability

The datasets generated during and/or analyzed during the current study are available from the corresponding author on reasonable request.
