# Supplementary material for: Case analysis of long-term negative psychological responses to psychedelics
Source: Sci Rep. 2023 Sep 25;13:15998. doi: 10.1038/s41598-023-41145-x (PMC10519946; doi:10.1038/s41598-023-41145-x)
Supplement: Supplementary file 1 — Supplementary Information 1. [file 41598_2023_41145_MOESM1_ESM.pdf]

Supplementary information

1. Case-by-case summaries

Case-by-case summary of interviewed participants (n=15). note, the cases numbers stated below are ordered according to whether the individual appears in the broader sample of 32. They therefore do not flow in a typical ordinal way e.g., participant 3 follows participant 1 etc.

Participant 1

Demographics: Male, aged 20 at time of experience.
Drug and dose: Psilocybin mushrooms, dose unknown, but described as "probably a lot".
Prior psychedelic experience: Once, one year prior to the currently relevant experience. He perceived the prior experience as "overall, a positive experience".
Relevant acute experience: Very frightening, including experiencing his own death - a suicide. Around 7.5 years had passed since the experience when interviewed.
Symptoms >72hrs after: Reported the occurrence or worsening of symptoms of depression or anxiety; extreme and problematic distractibility; impulsive behaviour; psychological distress; intrusive thoughts; anxiety; panic; sleep disturbances; complete loss of pleasure; extreme obsessive thoughts or behaviour; suicidal thinking; alienation/ isolation/ stigma; flashbacks of `bad trip`. Reported struggling with alcohol use after the experience.
Psychiatric history: Diagnosed with ADHD when he was around 4, Major Depressive Disorder (MDD) and chronic pain a few years before the experience (he could not remember exactly when). Struggling with binge eating for a few years (he was unsure of exactly when it started), but did not receive a formal diagnosis until after the psychedelic experience. Had withdrawn from ADHD medication in the months prior to the relevant experience.
Set & setting: With a group of people he did not know well. Had a sober sitter present. Had a personal intention of personal growth.

Participant 3

Demographics: Female, aged 27 at time of experience.
Drug and dose: LSD, 100 ug.
Prior experience of psychedelics: LSD twice, psilocybin once.
Relevant acute experience: Described as 'good' - until looking in the mirror – but after that, "very scary". Tried not to have a "bad trip". Approximately two years between the experience and interview.
Symptoms >72hrs after: Abnormally high mood; psychological distress; anxiety; panic; suicidal thinking; derealization; alienation/ isolation/ stigma; flashbacks of `bad trip`.
Psychiatric history: None prior to the psychedelic experience.
Set & setting: At home with boyfriend, felt uncertain about the relationship. This was the first time she had taken a psychedelic without a sober sitter.

Participant 4

Demographics: Female, aged 15 at time of experience.
Drug and dose: LSD, four tabs of two different kinds of LSD.
Prior experience of psychedelics: Used very regularly during the year before.
Relevant acute experience: Described feeling no effect first, doubled the dose, then it all started working and felt overwhelming. Interview took place 24 years after the experience.
Symptoms >72hrs after: Occurrence or worsening of symptoms of depression or anxiety; abnormally high mood; psychological distress; intrusive thoughts; anxiety; panic; auditory

hallucinations; suicidal thinking, planning or behaviour; visual perceptual aberrations; derealization; alienation/ isolation/ stigma; flashbacks of `bad trip`.
Psychiatric history: Diagnosed with bipolar disorder *after*, but believes she had the symptoms prior to use.
Set & setting: In town with a friend, "really wanted to get high". Later picked up by her mum's boyfriend and taken home.

Participant 7
Demographics: Male, aged 20 at time of experience.
Drug and dose: MDMA/ ecstasy, 12 pills – he thinks the total was around at least 800mg. Possibly cocaine and amphetamine the same evening too.
Prior experience of psychedelics: Regularly during several years. Used variety of drugs since age 13.
Relevant acute experience: Described as positive, although he did not feel as much of an increase as expected when redosing. Interview took place around 25 years after the experience.
Symptoms >72hrs after: occurrence or worsening of symptoms of depression or anxiety; psychological distress; intrusive thoughts; complete loss of pleasure; extreme obsessive thoughts or behaviours.
Psychiatric history: Diagnosed years later with MDD, bipolar and PTSD, although remembers having symptoms of all three prior to the MDMA-use. Aunt diagnosed with bipolar.
Set & setting: At a festival, stayed up all night.

Participant 10
Demographics: Female, aged 33 at time of experience.
Drug and dose: Ayahuasca, around 5 cups (i.e., about double the dose she had taken before).
Prior experience of psychedelics: Two series of 2 ayahuasca ceremonies during the year prior.
Relevant acute experience: Extreme nausea but unable to throw up during first ceremony. Second ceremony - nausea from the start. "My mind felt broken, I thought I would go insane." Interviewed roughly one year after the experience.
Symptoms >72hrs after: Extreme and problematic distractibility; psychological distress; intrusive thoughts; anxiety; panic; complete loss of pleasure; extreme obsessive thoughts or behaviours; derealization; alienation/ isolation/ stigma; flashbacks of the psychedelic experience.
Psychiatric history: None.
Set & setting: A series of two ayahuasca ceremonies, with a group of people and facilitated by a shaman. Reported possibly went into the second ceremony with a negative mindset "as if the ceremony before had primed it".

Participant 12
Demographics: Female, aged 28 at time of experience.
Drug and dose: LSD, "half a piece of paper", not sure about exact dose.
Prior experience of psychedelics: LSD once before (positive experience), MDMA many times.
Relevant acute experience: Felt unable to connect with boyfriend – thereafter spiralling into bad trip. Visual disturbances and misconception of time made her scared and uncomfortable. Interviewed around 3 months after the experience.

Symptoms >72hrs after: Occurrence or worsening of symptoms of depression or anxiety; psychological distress; intrusive thoughts; anxiety; panic; complete loss of pleasure; extreme obsessive thoughts or behaviours; alienation/ isolation/ stigma; flashbacks of bad trip.
Psychiatric history: Depression and SSRIs in the past, had recovered.
Set & setting: At home with boyfriend – had second thoughts about the relationship and intended to work on it with LSD. A lot of other life stresses and knew that she had to go to work next morning.

Participant 15
Demographics: Male, aged 36 at time of experience and his first time using (although this was not his first experience).
Drug and dose: Psilocybin mushrooms, and a little bit of MDMA the night before.
Prior experience of psychedelics: Psilocybin twice, around same dose, with experienced people.
Relevant acute experience: "…felt like a six hour long panic attack." Thought he would die. Interviewed four years after the experience.
Symptoms >72hrs after: Anxiety; panic; sleep disturbances; extreme obsessive thoughts or behaviours; alienation/ isolation/ stigma; flashbacks of the psychedelic experience.
Psychiatric history: Diagnosed with MDD and anxiety after, had symptoms before.
Set & setting: Shared the experience with a person who owed him money – tension between them, felt unsafe. The other person had no prior experience of psychedelics.

Participant 16
Demographics: Female, aged 30 at time of experience.
Drug and dose: MDMA/ ecstasy, 100 mg.
Prior experience of psychedelics: 2-5 times. First experience at aged 14.
Relevant acute experience: Overall good experience, describes it as not really knowing who she was and perhaps behaving inappropriately, which caused some tension in relationships. Interviewed around two months after the experience.
Symptoms >72hrs after: Occurrence or worsening of symptoms of depression or anxiety; extreme or problematic distractibility; psychological distress; intrusive thoughts; anxiety; sleep disturbances; paranoia; complete loss of pleasure; extreme obsessive thoughts or behaviours.
Psychiatric history: None.
Set & setting: At a party, someone she liked, and expected to soon start a relationship with, was present. Tension between them due to her behaviour on drugs.

Participant 20
Demographics: Female, aged 19 at time of experience and her first time using.
Drug and dose: Probably DMT – not sure as she were dosed against her will.
Prior experience of psychedelics: None.
Relevant acute experience: Extremely frightening and overwhelming. Reported being sexually abused while high. Interviewed around four years after the experience.
Symptoms >72hrs after: Occurrence or worsening of symptoms of depression or anxiety; extreme and problematic distractibility; psychological distress; intrusive thoughts; anxiety; panic; sleep disturbances; paranoia, delusional thinking; complete loss of pleasure; extreme obsessive thoughts or behaviours; addictive thoughts or behaviours; suicidal thinking, planning or behaviour; visual perceptual aberrations; derealization; flashbacks of the psychedelic experience.

Psychiatric history: Diagnosed with PTSD with psychotic features and BPD after the experience. Immediate family not diagnosed, but the participant believes that there is depression in her family.
Set & setting: With someone she saw as a friend, he gave her what she thought was cannabis and then sexually abused her when she was high.

Participant 22
Demographics: Female, aged 20 at time of experience.
Drug and dose: Psilocybin, ecstasy and cocaine. Unknown doses.
Prior experience of psychedelics: Psilocybin once before. First use at 16.
Relevant acute experience: Describes it as "very scary" – hallucinations of abuse and violence she had experienced growing up. Interviewed roughly twelve years after the experience.
Symptoms >72hrs after: Abnormally high mood; extreme and problematic distractibility; impulsive behaviour; psychological distress; intrusive thoughts; anxiety; panic; sleep disturbances; paranoia; extreme obsessive thoughts or behaviours; flashbacks of the psychedelic experience.
Psychiatric history: None.
Set & setting: With group of friends, travelling, recently ended a relationship, all friends in couples.

Participant 23
Demographics: Female, aged 38 at time of experience and her first time using.
Drug and dose: MDMA, 125 mg.
Prior experience of psychedelics: None.
Relevant acute experience: Describes as "mostly good and pleasant", though "scary" when the drugs started working. Interviewed around two years after the experience.
Symptoms >72hrs after: Occurrence or worsening of symptoms of depression or anxiety; psychological distress; intrusive thoughts; anxiety; panic; complete loss of pleasure; extreme obsessive thoughts or behaviours; self-harm; suicidal thinking; flashbacks of `bad trip`.
Psychiatric history: Depression and SSRIs ten years ago.
Set & setting: At home with sober boyfriend. The participant talked about having a negative bias towards drugs but wanted to try them in order to understand her boyfriend (who used drugs) better.

Participant 24
Demographics: Male, aged 28 at time of experience.
Drug and dose: LSD, one drop per hour from 2am to around 7 or 8 am.
Prior experience of psychedelics: Used almost every weekend during the two years prior.
Relevant acute experience: Physically sick, stomach pain, vividly hallucinating, hands turning blue. Describes it as "being pulled through a portal" and then paralyzed, unable to move anything else than his eyes, thought he was dead. Interviewed roughly 3.5 years after the experience.
Symptoms >72hrs after: Abnormally high mood; anxiety; panic; sleep disturbances; addictive thoughts or behaviours; visual perceptual aberrations.
Psychiatric history: Diagnosed with bipolar after, had symptoms before symptoms worsened after this.
Set & setting: At home, alone for most of it (partner at home during the first hours). Had woken up at 2am on a Saturday, after only 2-3 hours of sleep, "so excited" to try a new batch of LSD that he could not go back to sleep.

<u>Participant 25</u>
Demographics: Male, aged 21 at time of experience.
Drug and dose: LSD, 400ug (double his usual dose).
Prior experience of psychedelics: reports prior LSD-use "many times before" – describes "very positive" experiences.
Relevant acute experience: Described as "very positive [and] beautiful". Interviewed around two years after the experience.
Symptoms >72hrs after: Occurrence or worsening of symptoms of depression or anxiety; extreme and problematic distractibility; intrusive thoughts; anxiety; panic; sleep disturbances; delusional thinking; complete loss of pleasure; extreme obsessive thoughts or behaviours; visual perceptual aberrations; alienation/ isolation/ stigma.
Psychiatric history: Nothing formally diagnosed.
Set & setting: Alone at home, says things "felt good".

<u>Participant 26</u>
Demographics: Male, aged 21 at time of experience.
Drug and dose: LSD, 150-200ug.
Prior experience of psychedelics: Had used psychedelics 11-20 times before.
Relevant acute experience: Became frightening when one of the group had a "psychotic break". Interviewed roughly one year after the experience.
Symptoms >72hrs after: Intrusive thoughts; anxiety; panic; visual perceptual aberrations; alienation/ isolation/ stigma.
Psychiatric history: Not diagnosed, but experienced anxiety and `visual snow` prior – and this worsened after.
Set & setting: With a group of friends, one sober `sitter`.

<u>Participant 28</u>
Demographics: Nonbinary/ third gender, aged 15 at time of experience and first time using.
Drug and dose: LSD, 'one tab' i.e., estimated 75mcg.
Prior experience of psychedelics: None.
Relevant Acute experience: Became frightening when one of their friends "freaked out". The participant walked off into a forest alone. Interviewed nine years after the experience.
Symptoms >72hra after: Occurrence or worsening of symptoms of depression or anxiety; psychological distress; intrusive thoughts; anxiety; panic; sleep disturbances; paranoia; delusional thinking; auditory hallucinations; complete loss of pleasure; extreme obsessive thoughts or behaviours; suicidal thinking, planning or behaviour; visual perceptual aberrations; derealization; alienation/ isolation/ stigma; flashbacks of `bad trip`.
Psychiatric history: Diagnosed with depression with psychotic features (auditory hallucinations) prior.
Set & setting: With a group of friends at someone´s parents' house. Friend´s mother had tried to "kick them out" just at the time when the drugs started working. Felt very depressed in general.

2. Duration of the adverse psychological responses and potential sense of recovery

At the time of participation in this study, time since the psychedelic experience discussed ranged from 2 months to 25 years (mean = 6.8 years, SD = 8.5). Some participants considered themselves fully or mostly recovered when we spoke to them, but for most there was some ambivalence about the experience and whether or not the symptoms linked to it were still remaining or impacting their lives. For example, P25 and P26, who both had reported HPPD symptoms as their main adverse response, were still struggling when participating in the interview, around two years after the psychedelic experience. P26 believed their HPPD symptoms had subsided somewhat, and P25 described reduced anxiety about his HPPD over time, although the symptoms themselves had remained. P22 described reduced symptom severity (e.g., anxiety, agoraphobia, nightmares and flashbacks) over time, although her interview was 12 years after her experience, and she believed the experience had negatively impacted on her life for many years.

P20, P23 and P28 talked about their symptoms still being very impairing, although the latter had found psychotropic medication helpful and described severe mental health problems prior to the experience. P23, whose psychedelic experience was around two years prior to participation in this study, said that she still found it difficult to do everyday tasks and even struggled to get out of bed some days.

For those participants who considered themselves to be more or less recovered, the topic of what they believed to have helped in recovery came up naturally in interviews. Some participants had started taking psychotropic medication and thought that to have played an essential role in their improvement or recovery (P1, P15, P24). Some talked about social support (P1, P12) and lifestyle changes (P3, P12, P16, P22) being beneficial, one participant (P15) had found narrative-building around the experience important, and, perhaps unexpectedly, three participants (P3, P10, P16) had dealt with their prolonged adverse responses by using psychedelics again.

Another four participants (P4, P22, P25, P28) talked about how their symptoms had decreased with time. Five participants (P3, P15, P22, P24, P25) talked about experiencing personal growth after, and as a consequence of the challenging experiences discussed.

P20, P23 and P28 talked about their symptoms still being very impairing, although the latter had found psychotropic medication (Risperidone and Zoloft) helpful and described severe mental health problems prior to the experience. P23 still found it difficult to do everyday tasks and stayed in bed some days. Around two years had passed since her experience.

Five participants (P3, P15, P22, P24, P25) talked about experiencing personal growth after, and because of, their difficulties triggered by the psychedelic experience. This was, for example, described as “a recalibration to who I really am” (P15), finding motivation to change one’s life for the better (P25), and: “I felt [that] this period - in which I was having these derealization episodes and anxiety - was also a period of really intense personal growth” (P3). P22 said: “…and after this experience, I [was able to] see everything and I stopped smok[ing], … drink[ing], [and doing] cocaine. […] I don´t have any regrets. I do think that I learned something.”

3. Supplementary figure S1.

Visualisation of distribution of specific drug-use across 32 survey completers (left) and 15 interviewed participants (right). The category ‘Other’ included cannabis, risperidone

[prescribed anti-psychotic medication], and undefined drugs (i.e., participants ticked "other" without further explanation).

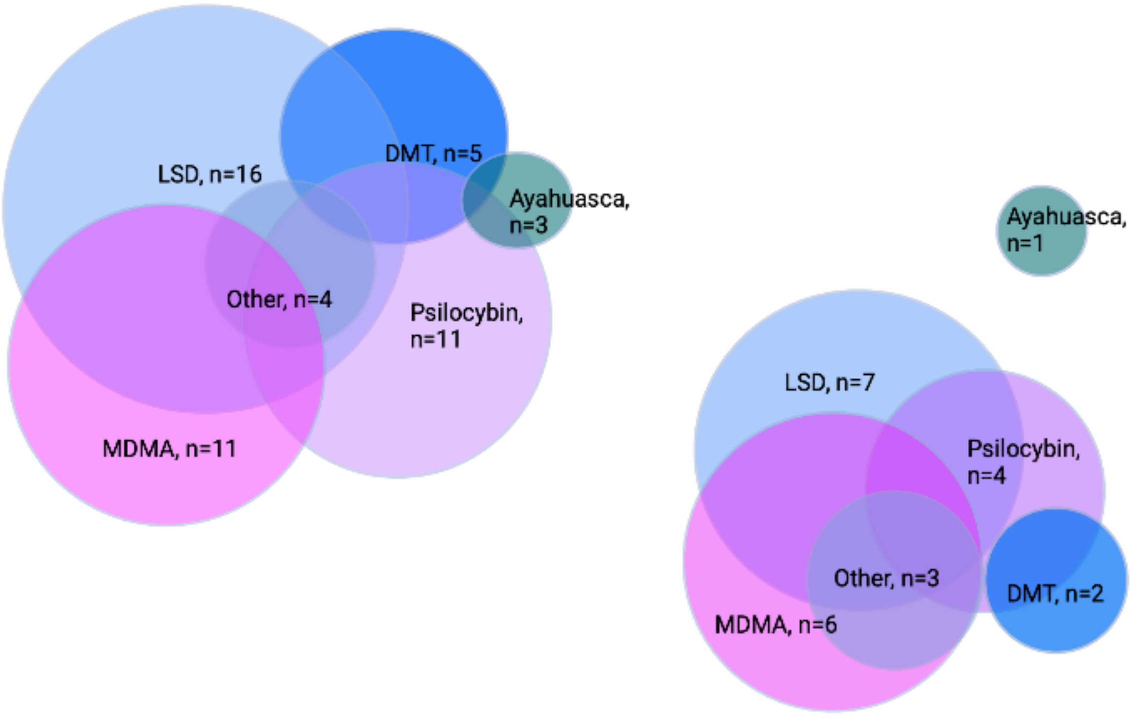

Supplementary figure S1: Visualisation of the whole sample of survey completers (N=32, on the left side) and interviewed participants (N=15, on the right side). The numbers presented in the picture refer to the number of participants reporting use of each drug in relation to the experience they felt had triggered their negative psychological response.

In the full sample of n=32, those who exclusively used single substances during their experience i.e., without polydrug mixing, were LSD n=11, psilocybin n=4, MDMA n=4, DMT n=1, ayahuasca n=2. No participant reported having used mescaline or 5-MeO-DMT during their experience. Thus, of the 32 survey responders, n=22 had used only one type of psychedelic, leaving n=10 who reported mixing substances.

Among the 15 interviewed participants, 11 reported exclusive use of just one drug during their experience: LSD n=6, psilocybin n=1, MDMA n=2, DMT n= 1, ayahuasca n=1. Of these, one person had recently lowered the dose of a stimulant for attention deficit hyperactivity disorder (ADHD) symptoms (Vyvanse) and stopped taking a non-stimulant (Strattera) for ADHD. Four other individuals reported some mixing of the listed psychedelics, ranging from 2-to-5 drugs mixed. Moreover, one of these individuals was currently using the prescribed anti-psychotic medication Risperidone.
