# Supplementary material for: Case analysis of long-term negative psychological responses to psychedelics
Source: Sci Rep. 2023 Sep 25;13:15998. doi: 10.1038/s41598-023-41145-x (PMC10519946; doi:10.1038/s41598-023-41145-x)
Supplement: Supplementary file 2 — Supplementary Information 2. [file 41598_2023_41145_MOESM2_ESM.docx]

*Supplementary Information Guide*

1. Case-by-case summary of interviewed participants
2. Duration of adverse psychological responses and potential sense of recovery
3. Supplementary figure 1: Visualisation of specific drug-use across 32 survey completers and 15 interviewed participants
